# Effect of periodontal therapy on hemoglobin and erythrocyte levels in chronic generalized periodontitis patients: An interventional study

**DOI:** 10.4103/0972-124X.51887

**Published:** 2009

**Authors:** Nupur Agarwal, Veerendra S. C. Kumar, Sheela A. Gujjari

**Affiliations:** 1 *PG Student, Department of Periodontics, J.S.S. Dental College & Hospital, Mysore, India*; 2 *Professor and Head, Department of Periodontics, J.S.S. Dental College & Hospital, Mysore, India*; 3 *Associate Professor, Department of Periodontics, J.S.S. Dental College & Hospital, Mysore, India*

**Keywords:** ACD, anemia, hemoglobin, periodontitis

## Abstract

**Aims and Objectives:**

Anemia of chronic disease (ACD) is one of the most common forms of anemia. It is defined as anemia occurring in chronic infections, inflammatory conditions or neoplastic disorders which are not due to marrow deficiencies or other diseases, and occurring despite the presence of adequate iron stores and vitamins. Periodontitis is one of the most prevalent chronic inflammatory diseases in humans. This study aimed at finding out if periodontitis, like other inflammatory conditions, could lead to anemia.

**Materials and Methods:**

Thirty chronic generalized periodontitis male patients with hemoglobin levels below 15 mg/dl and serum ferritin values above 30 ng/ml were selected. The various blood parameters recorded at baseline were hemoglobin levels(Hb), erythrocyte count (RBC), erythrocyte sedimentation rate (ESR), mean corpuscular volume(MCV), mean corpuscular hemoglobin (MCH) and mean corpuscular hemoglobin concentration (MCHC).

Periodontal parameters recorded at baseline included: plaque index, gingival index, probing pocket depth, clinical attachment level. Periodontal treatment including surgery if required was carried out in all the patients. Periodontal status of patients was monitored by repeating evaluation of periodontal indices at three months and at the end of one year. The hematological values were again measured at the end of one year.

**Results:**

The results showed that correction of periodontal inflammation resulted in a significant increase in hemoglobin levels and erythrocyte counts. The erythrocyte sedimentation rate showed a reduction indicating resolution of periodontal inflammation. There was a significant, but much lesser, improvement in MCV, MCH and MCHC values.

**Conclusion:**

The results of this study showed that treatment of periodontitis leads to an improvement in hematocrit and other related blood parameters in chronic generalized periodontitis patients with anemia. This provides evidence that periodontitis like other chronic diseases may also cause anemia.

## INTRODUCTION

The initiation and progression of gingivitis and periodontitis may be affected by certain systemic conditions. The converse side of the relationship between systemic health and oral health has also been demonstrated. This means that there may be potential effects of periodontal disease on a wide range of organ systems.[1]

Anemia of chronic disease is defined as anemia occurring in chronic infections, inflammatory conditions or neoplastic disorders that are not due to marrow deficiencies or other diseases, and occurring despite presence of adequate iron stores and vitamins.[2]

Recently, Hutter[2] have concluded that periodontitis, like other chronic conditions, lead to anemia. Other studies has failed to prove this relationship.[3] The present interventional study was carried out to evaluate periodontitis as one of the etiological factors leading to ACD.

## MATERIALS AND METHODS

Thirty male patients, aged between 30-55 years, with chronic generalized periodontitis were selected for the study. Patients chosen for the study had at least 30% or more of the teeth having greater than or equal to 4 mm probing depth and their hemoglobin levels were less than or equal to 15 mg/dl. Patients with systemic diseases; present and past smokers, patients who have undergone periodontal treatment six months prior to the study or who have less than 16 remaining teeth in the mouth were excluded from the study. Patients were included in the study only after they were explained the elected procedure in detail and gave their consent. The various hematological and clinical parameters were assessed at baseline.

### Hematological parameters

**Figure 1:**
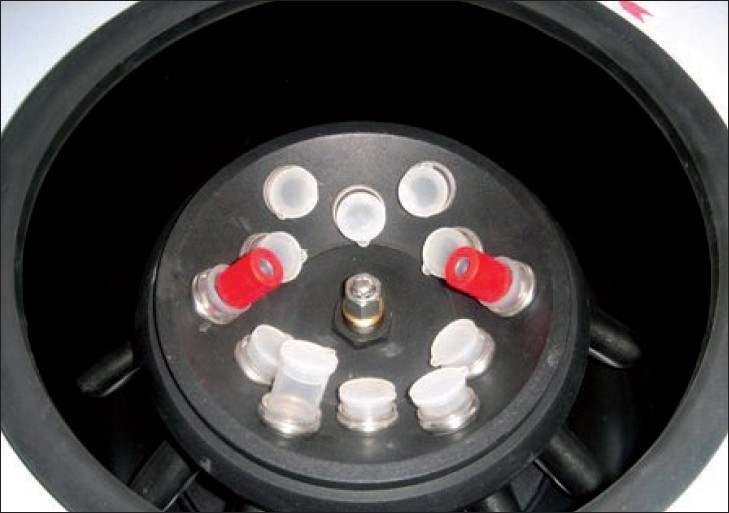
Withdrawal of blood for measuring serum ferritin, Hb, RBC, ESR and PCV at baseline

Under aseptic conditions, venous blood was drawn from ante-cubital fossa and sent for the following investigations [Figure 1].

**Figure 2:**
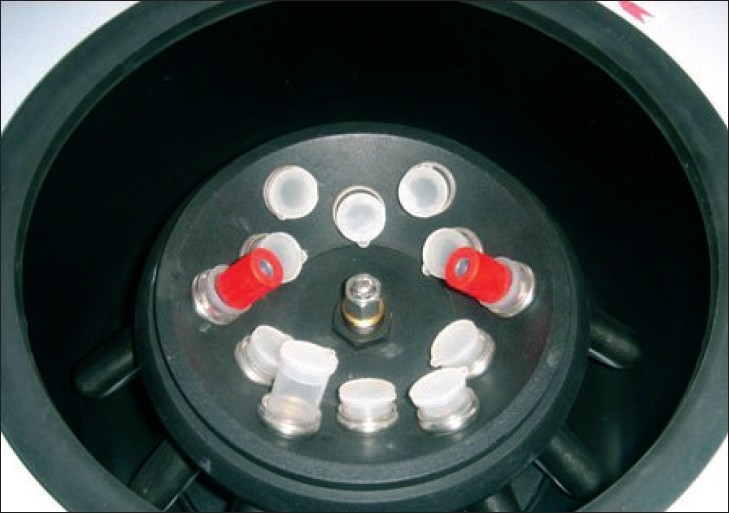
Centrifugation of blood using REMI R-8C laboratory centrifuge for measuring serum ferritin

Serum Ferritin was estimated at ANAND diagnostic laboratory, Bangalore. (NABL 15189 Accredited). Only patients with Serum ferritin above 30 ng/ml as well as hemoglobin values 15 mg/dl were included in the study. An inclusion of patients with serum ferritin value above 30 ng/ml was done to exclude patients with pure iron deficiency anemia [Figure 2].

**Figure 3:**
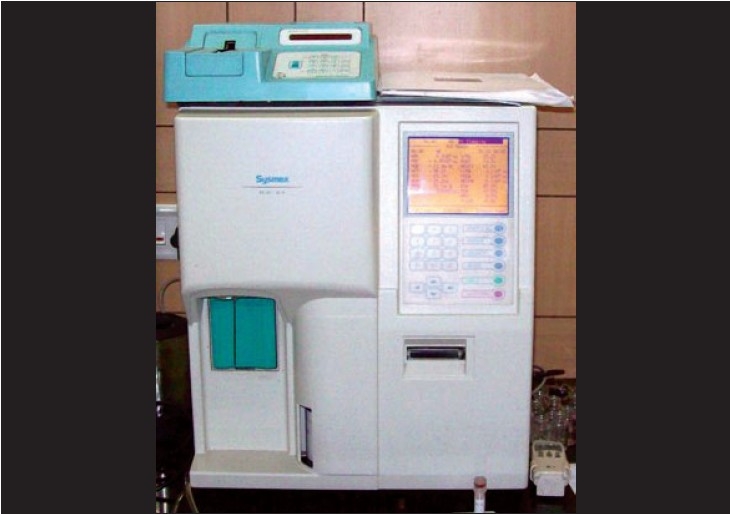
Symex cell counter used for measuring Hb, RBC and PCV

**Figure 4:**
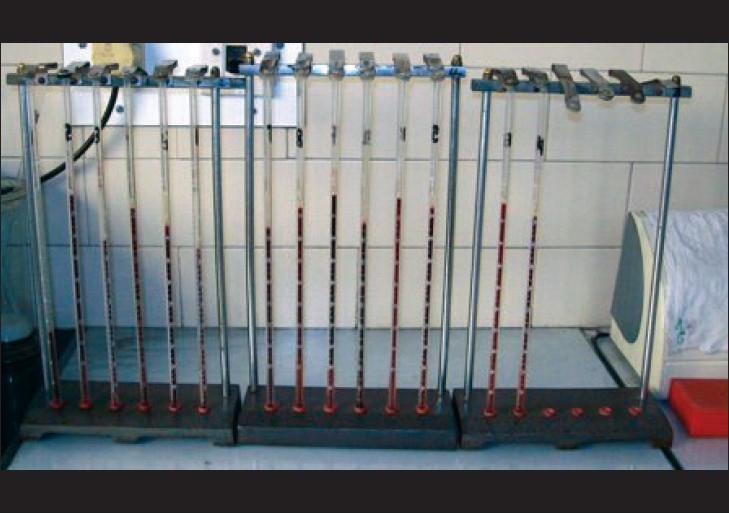
Westergren's method for measuring ESR

Other blood parameters estimated were [Figures 3 and 4]:

- Number of erythrocytes
- Hemoglobin concentration
- Erythrocyte sedimentation rate
- Mean Corpuscular volume
- Mean Corpuscular Hemoglobin
- Mean Corpuscular Hemoglobin concentration

### Clinical Parameters

**Figure 5:**
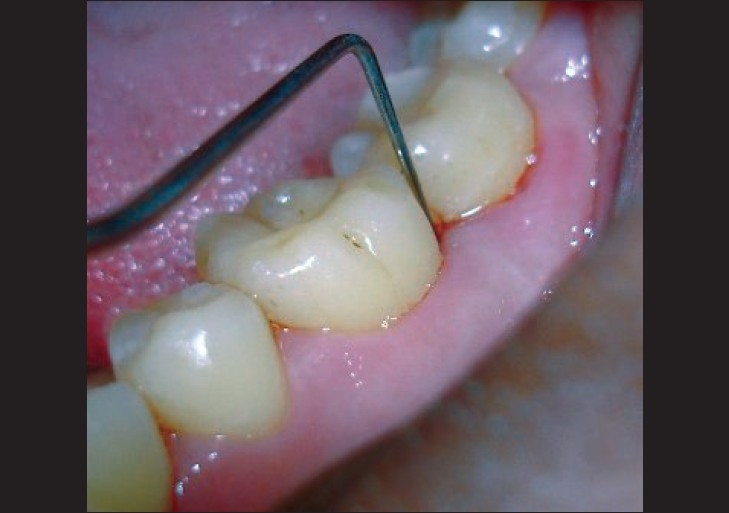
CPITN probe used for measuring the pocket depth and clinical attachment level at baseline

Detailed periodontal status of the patients included in the study was recorded using following *periodontal parameter* [Figure 5]:

- Plaque index according to Silness and Loe (1964).
- Gingival index according to Loe and Silness (1963).
- Probing pocket depth (Community Periodontal Index using CPITN probe).
- Clinical attachment level (Community Periodontal Index using CPITN probe).

**Figure 6:**
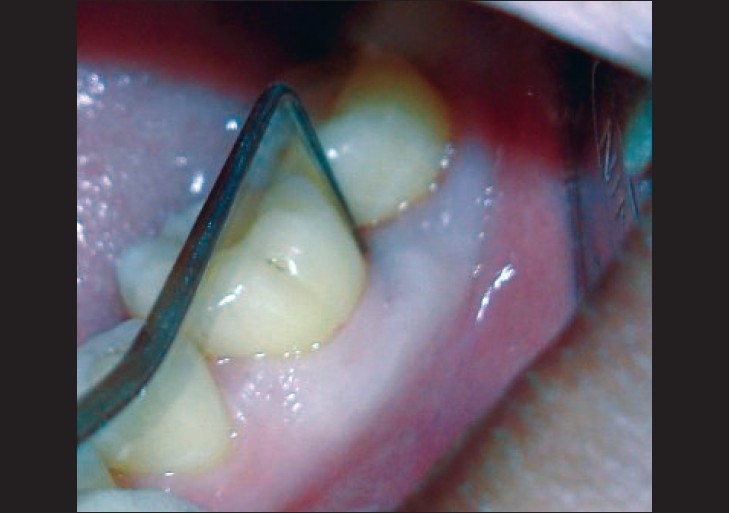
CPITN probe used for measuring the pocket depth and clinical attachment level at the end of 3 months

**Figure 7:**
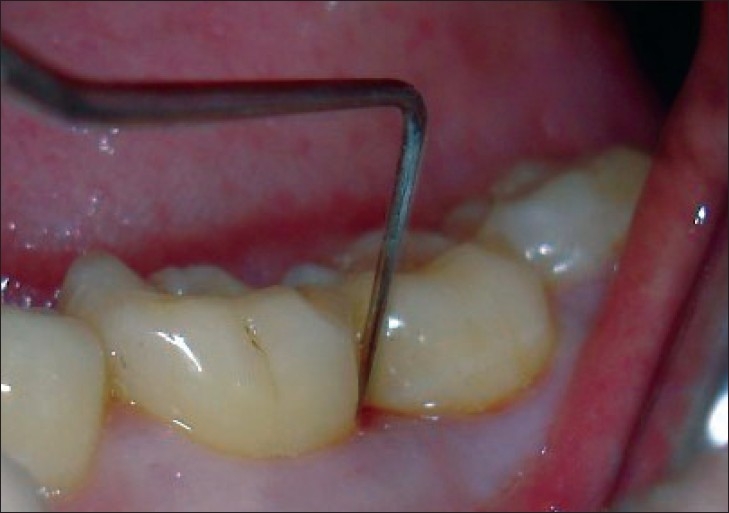
CPITN probe used for measuring the pocket depth and clinical attachment level at the end of 1 year

Full mouth scaling and root planing was carried out. Patients were not put on any iron or vitamin supplements. Neither was there any modification in the diet. After three months, periodontal indices were repeated. The CPI index was used in this study as it helped to decide the further treatment plan of the patient after three months [Figure 6]. All patients were put on maintenance phase. The periodontal indices and blood samples were again collected at the end of one year [Figure 7].

### Statistical Analysis

The collected data was subjected to statistical analysis through SPSS (Statistical Presentation System Software) for windows (version 10.0). The statistical methods applied were Paired sample‘t’ test, Repeated Measure ANOVA and Descriptive statistics. The paired Sample‘t’ test procedure was used to compare the means of two variables. Repeated Measures procedure provides analysis of variance and was used for the values got for clinical parameters. The results were presented in text, tables and graphs.

## RESULTS

**Graph 1:**
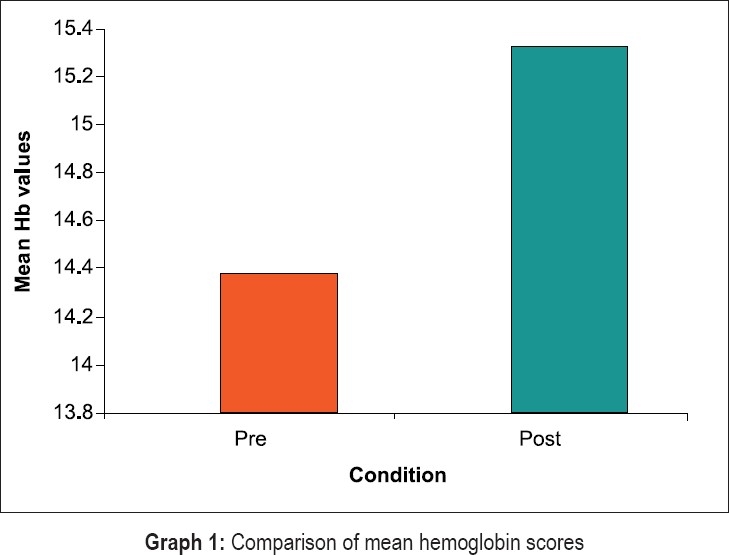
Comparison of mean hemoglobin scores

**Graph 2:**
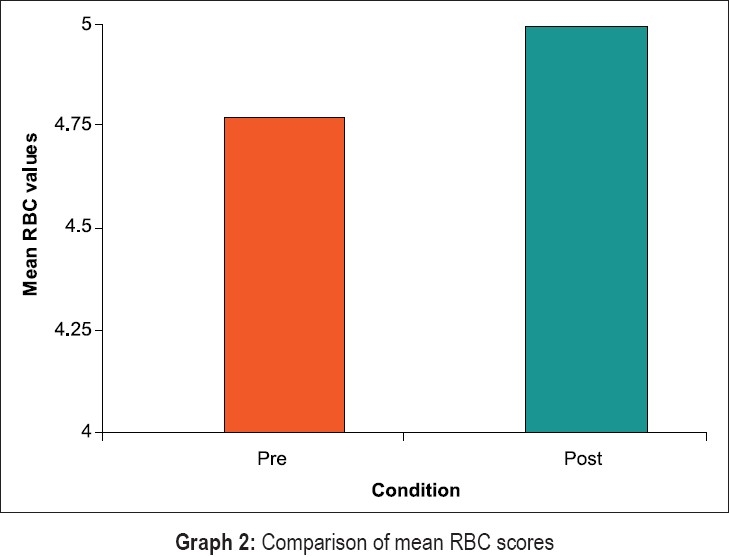
Comparison of mean RBC scores

**Graph 3:**
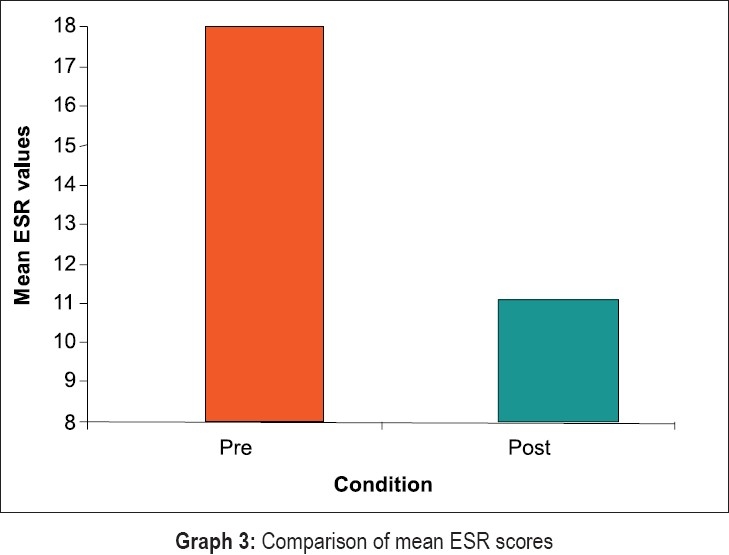
Comparison of mean ESR scores

**Graph 4:**
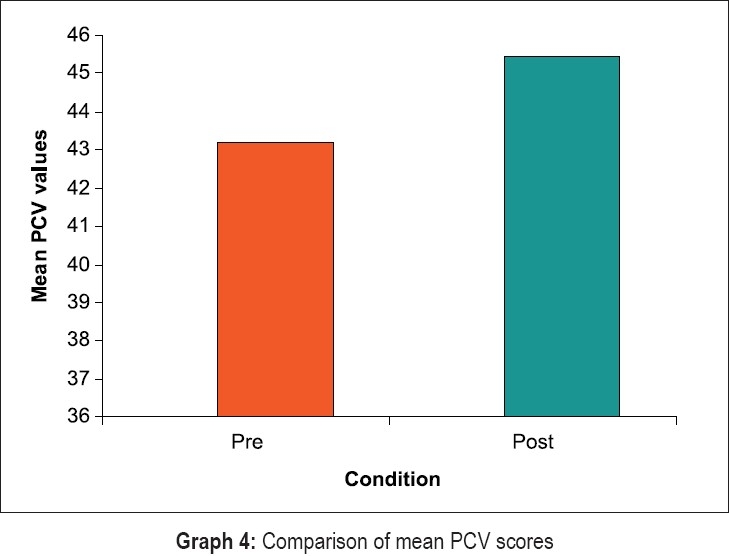
Comparison of mean PCV scores

Statistical evaluation of clinical and hematological parameters yielded the following results [Graphs 1–4]. Both Tables 1 and 2 show the mean and standard deviation of plaque scores at baseline, three months and at the end of one year. A statistically highly significant decrease from baseline to three months and a slight decrease from three months to one year were seen.

**Table 1:** Mean plaque index scores

	Mean	Std. deviation	N
PI baseline	1.6600	0.3233	30
PI 3 month	0.9167	0.2408	30
PI 1 year	0.8400	0.1380	30

**Table 2:** Repeated measures ANOVA for plaque index scores

Source	Type III sum of squares	Df	Mean square	F	Sig.
Change	12.308	2	6.154	144.406	.000
Error (change)	2.472	58	4.262	-	-

Tables 3 and 4 show the mean and standard deviation of calculus scores at baseline, three months and end of one year. A statistically highly significant change was observed from baseline to three months and slight change from three months to one year. F value was found to be significant at 0.000 levels.

**Table 3:** Mean gingival index scores

	Mean	Std. deviation	N
GI baseline	1.7433	0.1924	30
GI 3 month	1.0300	0.1878	30
GI 1 year	0.8000	0.1597	30

**Table 4:** Repeated measures ANOVA for gingival index scores

Source	Type III sum of squares	Df	Mean square	F	Sig.
Change	14.516	2	7.258	615.654	.000
Error (change)	0.684	58	1.179	-	-

In Tables 5 and 6, the mean values and standard deviations for CPI score for probing pocket depth at baseline, three months and end of one year are presented. No change was observed from baseline to three months. A statistically significant change was observed from three months to one year

**Table 5:** Mean CPI pocket probing depth scores

	Mean	Sth. deviation	N
PPD baseline	4.0000	0.0000	30
PPD 3 month	4.0000	0.0000	30
PPD 1 year	3.2667	0.0000	30

**Table 6:** Repeated measures ANOVA for probing pocket depth scores

Source	Type III sum of squares	Df	Mean square	F	Sig.
Change	10.756	2	5.378	39.427	0.000
Error (change)	7.911	58	0.136	-	-

In Tables 7 and 8, the mean values and standard deviations for CPI score for clinical attachment level at baseline, three months and end of one year are presented. When the data was subjected to repeated measures ANOVA, no change was observed from baseline to three months. Significant difference was seen between three months to one year

**Table 7:** Mean CPI clinical attachment level scores

	Mean	Std. deviation	N
CAL baseline	2.6000	0.6747	30
CAL 3 month	2.6000	0.6747	30
CAL 1 year	2.0333	0.6687	30

**Table 8:** Repeated measures ANOVA for CPI clinical attachment scores

Source	Type III sum of squares	Df	Mean square	F	Sig.
Change	6.422	2	3.211	37.923	.000
Error (change)	4.911	58	8.467	-	-

In Table 9, results for the overall data analyses for hematological parameters are presented. The hemoglobin, erythrocyte count and hematocrit values were significantly increased post treatment at the end of one year (p less than 0.000). Further, the ESR values were higher at the end of one year (p less than 0.000). The increase in values of MCV, MCH and MCHC post treatment in our study was not very high.

**Table 9:** Paired-samples ‘t’ test - Hematological values

	Mean	Std. deviation	Mean change	‘t’ value	Significance (p)
Hb					
Baseline -	14.38	0.48	0.9533	12.684	0.000
1 year	15.333	0.5397			
RBC					
Baseline -	4.777	0.441	0.2200	9.485	0.000
1 year	4.9967	0.4343			
ESR					
Baseline -	18.47	6.23	7.3667	8.004	0.000
1 year	11.1000	2.2644			
PCV					
Baseline -	43.20	1.71	2.2667	10.333	0.000
1 year	45.4667	1.7367			
MCV					
Baseline -	90.8433	5.7549	0.7027	4.258	0.05
1 year	91.5460	5.4629			
MCH					
Baseline -	30.257	2.100	0.6043	7.248	0.05
1 year	30.8610	2.0150			
MCHC					
Baseline -	33.31	0.82	0.4007	5.598	0.05
1 year	33.7073	0.9393			

The overall results of hematological parameters as shown in Table 9 are represented by bar graphs.

## DISCUSSION

The present interventional study was carried out to measure the effect of periodontal therapy on hemoglobin and erythrocyte levels in chronic generalized periodontitis patients with anemia.

In an evaluation by Merchant,[4] the clinical implication of the study by Hutter[2] was said to be limited because of potential residual confounding by smoking, failure to show the association among non or past smokers, and failure to adjust for menopausal status in women. To do away with these drawbacks, only males were included in this study. Further, both past and present smokers were excluded to remove any confounding effect of smoking. In our study, we included 30 chronic generalized periodontitis male patients with hemoglobin less than or equal to 15 gm/dl to evaluate effect of periodontal therapy on blood parameters. A lower cut off hemoglobin value was not kept as studies have shown that there were no significant differences in frequencies of periodontitis patients with red blood cell parameters below or above the laboratory reference values.[2,5] Also, the effort was to include patients with severe generalized periodontitis. The reasoning behind this was the observation of a correlation between the degree of anemia and the severity of the underlying disease.[6] Only patients with serum ferritin above 30 ng/ml were included in the study. This was done to exclude patients with pure iron deficiency anemia.[7]

The relation between anemia and periodontitis has been explored in the latter half of 20^th^ century. The studies investigated both the concept of anemia as an etiological factor of periodontitis and periodontitis as a risk factor of anemia. It was hinted and believed that anemia may be a factor in the causation of periodontitis rather than a consequence. Chawala[8] suggested anemia as an important factor in the causation or pathogenesis of periodontal disease. Lainson also put forward anemia as a systemic cause of periodontitis. In 1935, the concept that the depression in the number of erythrocytes is apparently secondary to the presence of periodontal disease was first put forward clearly by Epstein. He reached to this conclusion as arrest or cure of chronic periodontal disease in his patients resulted in the elevation of the erythrocyte count to normal or high normal levels.[2,8] Siegel[9] reported a depression in the number of erythrocytes apparently secondary to the presence of periodontal disease, since arrest or cure of these pathological processes in individual cases resulted in the elevation of the erythrocyte count to normal or high normal levels. Chronic periodontal disease as a possible contributing factor or cause of mild anemia was also indicated.

Recently, various studies have tried to evaluate the relation between periodontitis and anemia. These studies present conflicting results. Hutter[2] and Thomas[5] showed that periodontitis patients have a lower hematocrit, lower numbers of erythrocytes, lower hemoglobin levels and higher erythrocyte sedimentation rates. Whereas, the studies by Wakai[3] and Havemose-poulsen A[10] failed to show any association between hemoglobin levels and periodontal status. A ten week intervention study.[11] found an increase after scaling and root planing (SRP) in hemoglobin and RBC levels in patients with severe periodontitis. A statistically significant (p less than 0.05) increase from the mean hemoglobin level of 14.5 mg/dl at baseline to 15 mg/dl at 10 weeks after SRP was seen.

All of these studies, except one, are epidemiological in nature. We carried out an interventional study as it is a better design to identify risk factors for any disease of interest.[1] Our study showed a change in mean hemoglobin value by 0.9533 mg/ dl (*P* less than 0.05) and a mean increase of 0.22 million/mm^3^ in erythrocyte count on resolution of periodontal inflammation. These changes are in agreement with a previously reported interventional study.[11] Slightly more improvement in the present study may be due to longer time period of one year in which surgery was carried out if required. The finding that hemoglobin, RBC and other hematocrit values increased after periodontal treatment is also supported by recent observational studies reporting a difference in these values between periodontitis patients and control.[2,5] The increase in values of MCV, MCH and MCHC post treatment in our study was not very high. The minimal change in MCV indicates that the anemia due to periodontitis is normocytic and hence not due to iron or vitamin deficiency. Again, the small increment in MCH and MCHC values compared to increase in hemoglobin levels implies that anemia associated with periodontitis is of normochromic type.[12]

The change in hemoglobin and RBC values in our study is statistically highly significant but not as high as observed in anemia due to other inflammatory conditions like rheumatoid arthritis[13] and multiple myeloma.[14] This might be due to the reason that the other diseases are more severe inflammatory conditions than periodontitis. So anemia resulting from periodontitis is relatively mild. This also might be the reason why few studies[3] failed to find any relation between hematocrit indices and periodontal status.

The current study also showed a decrease in ESR values after treatment. The determination of ESR is helpful in assessing the progress of patients treated for certain chronic inflammatory disorders. It has been reported to decrease after periodontal treatment.[2]

The present study establishes that periodontitis leads to a mild anemia with a decrease in Hb and RBC values. Proinflammatory cytokines such as TNF-α, IL-1β, INF-γ and PGE_2_ are found in high concentrations in inflamed periodontal tissues. The various cytokines can enter the blood circulation and affect distant sites and organs.[1] The same inflammatory cytokines have been found to be central in the pathogenesis of ACD.[6,7] So, it can be concluded that periodontitis, like other chronic inflammatory conditions, leads to ACD.

## CONCLUSION

The aim of the present study was to assess the effect of periodontal therapy on hematological values and thus anemic condition in patients with severe chronic generalized periodontitis. The following conclusions were arrived at from this study:

1. Hemoglobin value, erythrocyte count, MCV, MCHC and MCHC levels showed a significant improvement after periodontal treatment.
2. As there was no other diet modification or prescribed supplements, the improved hematological values indicated that periodontitis should be considered as one of the conditions causing anemia of chronic disease.
3. The improvement observed in mean hemoglobin levels after therapy in chronic generalized periodontitis patients was 0.9533 mg/ dl. It implied that periodontitis leads to significant but not severe decrease in hemoglobin values. Thus, ACD resulting from periodontitis should not be expected to be severe.

However this study had a drawback of limited sample size. Further, a relation between severity of periodontitis and anemia was not explored in the present study. Further studies with larger sample size are warranted.
